# High visual demand following theta burst stimulation modulates the effect on visual cortex excitability

**DOI:** 10.3389/fnhum.2015.00591

**Published:** 2015-10-28

**Authors:** Sabrina Brückner, Thomas Kammer

**Affiliations:** Section for Neurostimulation, Department of Psychiatry, University of UlmUlm, Germany

**Keywords:** cortical excitability, phosphene threshold, theta burst stimulation, transcranial magnetic stimulation, visual cortex

## Abstract

Modulatory effects of repetitive transcranial magnetic stimulation (TMS) depend on the activity of the stimulated cortical area before, during, and even after application. In the present study, we investigated the effects of theta burst stimulation (TBS) on visual cortex excitability using phosphene threshold (PTs). In a between-group design either continuous or intermittent TBS was applied with 100% of individual PT intensity. We varied visual demand following stimulation in form of high demand (acuity task) or low demand (looking at the wall). No change of PTs was observed directly after TBS. We found increased PTs only if subjects had high visual demand following continuous TBS. With low visual demand following stimulation no change of PT was observed. Intermittent TBS had no effect on visual cortex excitability at all. Since other studies showed increased PTs following continuous TBS using subthreshold intensities, our results highlight the importance of stimulation intensity applying TBS to the visual cortex. Furthermore, the state of the neurons in the stimulated cortex area not only before but also following TBS has an important influence on the effects of stimulation, making it necessary to scrupulously control for activity during the whole experimental session in a study.

## Introduction

Applying pulses of transcranial magnetic stimulation (TMS) to the occipital pole can result in elementary visual percepts called phosphenes (Barker et al., 1985; Meyer et al., 1991; Marg and Rudiak, 1994). The phosphene threshold (PT), the minimum TMS intensity which elicits phosphenes in a single subject, indicates the individual excitability of the stimulated cortex area. Quite a few studies have stated that PT is a stable parameter for visual cortex excitability, showing low test-retest-variability within the same subject (e.g., Boroojerdi et al., 2000a, 2002; Stewart et al., 2001; Antal et al., 2003; Siniatchkin et al., 2011). PT systematically varies with different TMS pulse forms (monophasic vs. biphasic) similar to variations known from the motor cortex, indicating that the physiological processes induced in the visual system are comparable to those observed in the motor system (Kammer et al., 2007; Kammer and Baumann, 2010).

Using PT as dependent variable, various studies investigated changes in visual cortex excitability under certain conditions. For instance, Boroojerdi et al. (2000a) showed reduced PTs following light deprivation, indicating a substantial increase in visual cortex excitability. In contrast to such a binocular deprivation, it was shown that monocular deprivation acutely triggers a reversible decrease in visual cortex excitability (Lou et al., 2011). As a neurophysiological correlate for clinical observations inferring hyperexcitability of the occipital cortex, lower PTs were shown in migraineurs compared to normal controls (Aurora et al., 2003). Demonstrating increased visual cortex excitability, lower PTs were shown following a period of reading (Antal et al., 2014). Thus, measuring PTs applying single pulses of TMS is a common tool to investigate changes in visual cortex excitability.

Besides determining cortical excitability TMS is also able to modulate excitability if applied repetitively (rTMS). In the visual cortex, PT is often used to calibrate stimulation intensity for modulating visual cortex functions by rTMS. Furthermore, PT serves as dependent variable in order to investigate the effects of such a modulation. Thus, several studies showed that 1 Hz rTMS can increase PTs in healthy subjects by probably reducing cortical excitability (Boroojerdi et al., 2000b; Brighina et al., 2002; Fierro et al., 2005). Additionally, rTMS differentially modified the effects of light deprivation on PTs depending on stimulation frequency (Fierro et al., 2005). As shown by Franca et al. (2006) theta burst stimulation (TBS), a particular rTMS protocol (Huang et al., 2005), applied with 80% of the individual PT intensity is also able to modulate PTs. Whereas increased PTs were found following continuous TBS (cTBS), intermittent TBS (iTBS) showed no modification of PTs (Franca et al., 2006).

The effects of TMS on brain functions depend on different factors such as coil geometry (Fleming et al., 2012), pulse waveform (Kammer et al., 2001b), current direction (Kammer et al., 2001a) or other external parameters (Sandrini et al., 2011). Moreover, internal factors may influence the rTMS induced modulations. For instance, there is evidence that the initial state of neurons undergoing TMS plays an important role for the aftereffects (Silvanto and Pascual-Leone, 2008). In the visual cortex, Silvanto et al. (2007) showed the state-dependency of offline high-frequency TMS by selective manipulation of network subareas via adaptation on colored stimuli. Furthermore, various studies investigated the effects of different brain stimulation techniques in dependency of previous activity in the same network (Müller-Dahlhaus and Ziemann, 2015), indicating that metaplasticity indeed is operative in brain functions. For instance, there is evidence for homeostatic plasticity in the motor cortex (Siebner et al., 2004) as well as in the visual cortex (Bocci et al., 2014). In both studies, the initial state of the brain area was first selectively manipulated using transcranial direct current stimulation. The subsequent rTMS protocols showed different effects on cortex functions, depending on the induced activity in the system before, respectively. Apart from preconditioning of the cortex before rTMS, enhanced activity even after rTMS can modify the effect. In a motor cortex study, contraction of the target muscle immediately after TBS enhanced the facilitatory effect of iTBS and reversed the inhibitory effect of cTBS into facilitation (Huang et al., 2008). Besides that, it is known from several studies that visual demand influences visual cortex excitability (Rauschecker et al., 2004; Antal et al., 2014). Thus, controled visual demand following TBS applied over the visual cortex might be able to modulate the TBS effects as observed in the motor system (Huang et al., 2008).

In the present study, we set out to investigate the effects of both cTBS and iTBS on the excitability of the visual cortex (between-group design). Since higher TBS intensities might increase the size and duration of the modulation (Brückner et al., 2013), we chose 100% of the individual PT as intensity for TBS. Additionally, as hypothesized in our previous work (Brückner and Kammer, 2014), we examined whether enhanced activity in the visual cortex caused by a visual acuity task following TBS might influence the TBS modulation. In a within-design subjects underwent two sessions, with and without an enhanced demand of the visual cortex following TBS, respectively.

We expected the following effects: (a) TBS will modify PT; (b) the modulation of PT will last for some time if subjects have low visual demand following TBS; and (c) the modulation will be affected if subjects have high visual demand following TBS.

## Materials and Methods

### Subjects

In total, 53 subjects were recruited for the study. Exclusion criteria were metallic implants, prior history of neurological or psychiatric disorders, chronic tinnitus, major medical illness, drug abuse or alcoholism, any medication with the exception of contraceptives. Thirteen subjects were excluded due to the following reasons: four subjects gave no written informed consent, eight subjects showed instability of baseline PT and one subject fell asleep during the measurement. The remaining 40 subjects were divided into two groups (20/20) receiving either cTBS (mean age 24.1 ± 3.8 years, 10 male) or iTBS (mean age 23.0 ± 2.6 years, 10 male). All subjects had normal or corrected-to-normal visual acuity as revealed by the Freiburg Visual Acuity Test (FRACT; Bach, 1996). All participants gave their written informed consent and were paid for participation. The study followed the Declaration of Helsinki and was approved by the local ethics committee.

### Experimental Design

Subjects sat in a comfortable chair during the whole session. Biphasic magnetic pulses were delivered using a Magpro X100 stimulator (MagVenture Farum, Denmark) and a figure-of-eight-coil (MC-B70). The coil position in relation to the head was monitored, registered, and maintained using the frameless stereotactic positioning system BrainView (V2, Fraunhofer IPA, Stuttgart, Germany, cf. Kammer et al., 2007).

In their first session, subjects started with a familiarization procedure where they were trained to observe phosphenes. Approximately 2 cm above the inion, the coil was held tangentially to the skull and single pulses were applied starting with an intensity of 30% of maximum stimulator output (MSO). Intensity was increased in steps of 5% until the subject either perceived a phosphene or an intensity of 70% MSO was reached without any percept. In this case, the coil was moved in steps of about 5 mm over the occipital pole and the procedure was repeated until a phosphene was perceived. Phosphene perception had to fulfill the following criteria (cf. Kammer et al., 2005): (a) dependency on the stimulated hemisphere, i.e., perception in the left visual field with stimulation at the right occipital pole and* vice versa* (Meyer et al., 1991); (b) visibility with both eyes open or closed (Kammer and Beck, 2002); and (c) dependency on gaze direction (Meyer et al., 1991). Following the familiarization procedure, the coil position inducing the strongest percept was located. Then the coil was rotated in order to determine the optimal current direction (Kammer et al., 2005). In most cases, the final coil position (“hot-spot”) was located on the left hemisphere, inducing a current in latero-medial direction. PTs were then measured following a previously established protocol (Kammer et al., 2001a): 49 magnetic pulses were delivered at seven different stimulator output intensities in steps of 3% with at least 5 s in between. Intensities were randomly intermixed and each intensity was applied seven times (method of constant stimuli). Intensity range was defined on the basis of the approximate individual threshold known from the familiarization procedure. Subjects had to press one of two buttons indicating whether they have perceived a phosphene or not. A sigmoidal fit applied to the responses generated the individual PT at the reversal point of the logistic function (psignifit; Wichmann and Hill, 2001).

**Figure 1:**
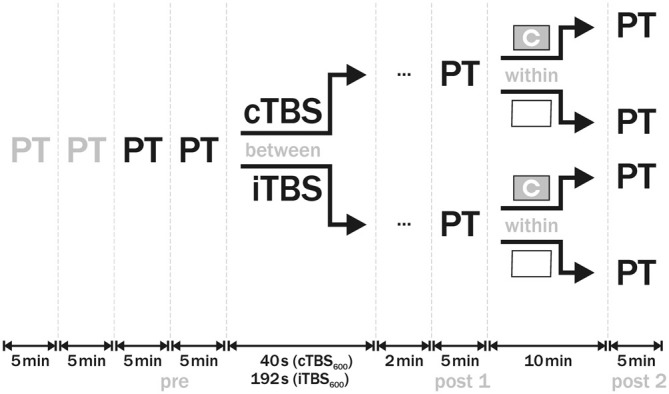
**Design of the study.** At the beginning of each session, phosphene threshold (PT) was measured four times. The first two measurements were discarded as practice runs, the other two were averaged as baseline (pre) PT value. In a between-group design, either cTBS or iTBS was then applied to visual cortex with 100% PT intensity. Two minutes after the end of theta burst stimulation (TBS), PT (post 1) was established. In the subsequent 10 min, subjects either completed a Landolt C optotype task in the one session or kept looking at a white wall in the other session. Afterwards, PT was measured again (post 2). Order of sessions was counterbalanced across subjects.

In each session, PT was initially measured four times: two practice runs (discarded) and two baseline measurements (mean as individual “pre” PT value). Then, either cTBS or iTBS was applied to the predetermined phosphene hot-spot. TBS intensity was set to 100% of individual PT. Two minutes after the end of stimulation, PT was estimated again (post 1) using the same method and intensities as in the baseline measurements. For the next 10 min, in a within-design subjects had either low or high visual demand. In the low visual demand session, they were instructed to keep their eyes open looking at a white wall. In the high visual demand session, subjects performed a visual task (see below). Following the visual demand period, PT was estimated again (post 2). Order of sessions was counterbalanced across participants, with at least 48 h in-between. The design of the study is illustrated in Figure 1.

The visual task consisted of visual acuity measurements using Landolt C optotypes. It was realized in PsychoPy (v.1.70, Peirce, 2007) on a standard personal computer and a cathode ray tube computer monitor (21″, iiyama Vision Master Pro 500, iiyama, Tokyo, Japan) with a frame rate of 100 Hz and a resolution of 800 × 600 pixels. A red fixation point was presented in the middle of the screen for 1000 ms followed by a central Landolt C optotype with its gap oriented either up, down, right or left. The target was presented for 10 ms followed by a mask consisting of random noise pixels. Subjects had to indicate the direction of the gap using the arrow keys on a standard keyboard. The size of the Landolt C optotype was varied following a simple 2:1 staircase procedure. The variation occurred in steps of one pixel, starting with a gap of 10 pixels. After seven reversals of the staircase, the run was terminated and another run was started to avoid fatigue. A single run of the task lasted 1.5–2 min, thus subjects had to complete 5–6 runs within the 10 min. Visual acuity thresholds were not analyzed further, the visual task was only used for induction of high visual demand.

### Data Analysis

The four baseline PT values from the participants (2 sessions × 2 baseline values) were analyzed with regard to stability using a repeated-measures analysis of variance (rmANOVA). Pre and post TBS PTs were subjected to an rmANOVA for each group separately (Statistica V.10, StatSoft GmbH, Hamburg, Germany). Sphericity requirements were assessed using Mauchley’s test. Sphericity assumption was not violated in any test, thus correction was dispensable. *Post hoc* analyses were performed using Newman-Keuls test.

## Results

### Baseline Values

Baseline PT value was measured four times at the beginning of each session. Whereas the first two measurements were taken as practice runs and discarded, the other two measurements were analyzed due to stability of PTs.

The two baseline values in each of the two sessions were subjected to an omnibus rmANOVA with the between-factor GROUP (cTBS/iTBS) and the within-factors MEASUREMENT (1 and 2) and SESSION (high/low visual demand). It revealed that there was no main effect on any condition and no interaction. *F*- and *p*- values are reported in Table 1.

**Table 1:** **Analysis of baseline stability**.

Effect	*F*_(1, 38)_	*p*
GROUP	3.809	0.058
SESSION	0.060	0.807
MEASUREMENT	1.176	0.285
SESSION × GROUP	0.444	0.509
MEASUREMENT × GROUP	1.154	0.289
SESSION × MEASUREMENT	1.009	0.322
SESSION × MEASUREMENT × GROUP	0.674	0.417

Due to stability in baseline values, the two baseline measurements of each participant in a session were averaged as pre-TBS PT value. Baseline PT values of the iTBS group were numerically higher than those of the cTBS group (*F*_(1, 3)_ = 3.81, *p* = 0.058). For sake of clarity, we analyzed the data of the two groups separately.

### cTBS

**Figure 2:**
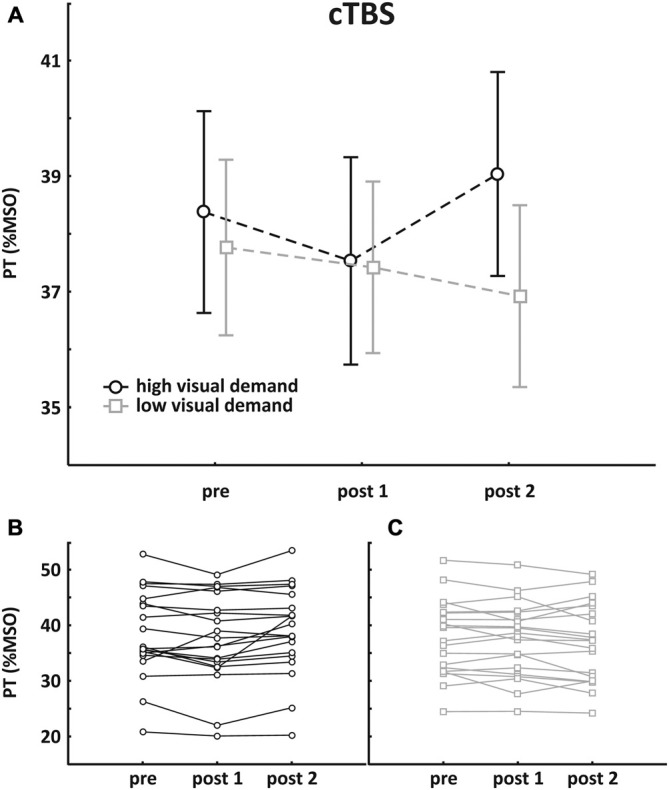
**Results of the cTBS group. (A)** Mean group PT change (±SEM); **(B)** individual data with high visual demand; **(C)** individual data with low visual demand. An rmANOVA with the within-factors SESSION (high/low visual demand) and TIME (pre, post1, post2) revealed no main effect, but a significant interaction was found, indicating that PTs increased following high visual demand preceded by cTBS.

Mean pre-TBS PT value was 38.4 ± 7.8% MSO in the session with high visual demand and 37.8 ± 6.8% MSO in the session with low visual demand, respectively. Pre- and post-TBS PTs were subjected to an rmANOVA with the within-factors SESSION (high/low visual demand) and TIME (pre, post1, post2). No main effect was found (SESSION: *F*_(1, 19)_ = 0.92, *p* = 0.35; TIME: *F*_(2, 38)_ = 1.95, *p* = 0.16) but the interaction of the factors was significant (*F*_(2, 38)_ = 4.61, *p* = 0.016). *Post hoc* analysis revealed that cTBS had no direct effect on PT but an increased PT was found after the visual acuity task. Mean group data as well as individual data are depicted in Figure 2.

### iTBS

**Figure 3:**
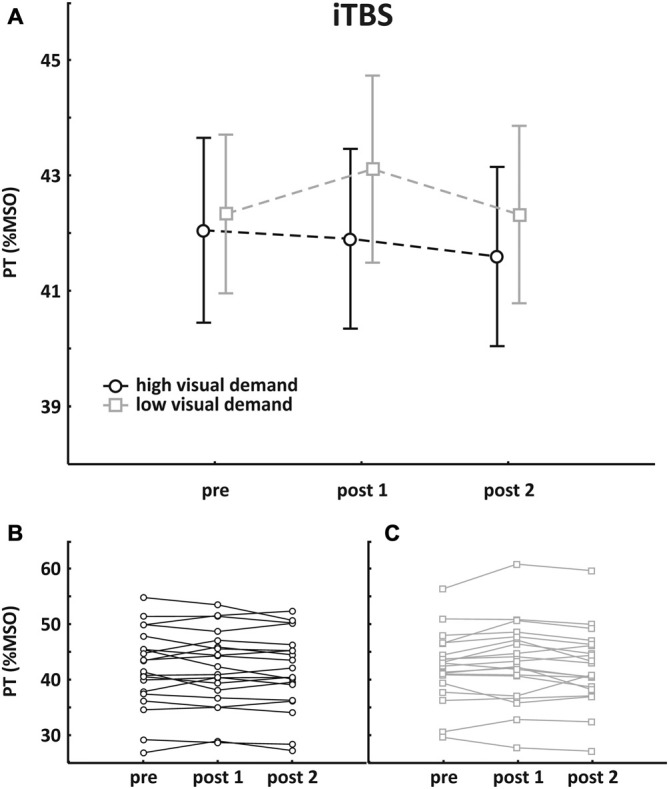
**Results of the iTBS group. (A)** Mean group PT change (±SEM); **(B)** individual data with high visual demand; **(C)** individual data with low visual demand. An rmANOVA with the within-factors SESSION (high/low visual demand) and TIME (pre, post1, post2) revealed no main effect and no interaction.

Mean pre-TBS PT value was 42.1 ± 7.2% MSO in the session with high visual demand and 42.3 ± 6.2% MSO in the session with low visual demand, respectively. Pre- and post-TBS PTs were subjected to an rmANOVA with the within-factors SESSION (high/low visual demand) and TIME (pre, post1, post2). There was no main effect (SESSION: *F*_(1, 19)_ = 0.76, *p* = 0.39; TIME: *F*_(2, 38)_ = 1.61, *p* = 0.21) and no interaction. Mean group data as well as individual data are depicted in Figure 3.

### External Factors

To evaluate whether there are possible effects of age, gender or order of the sessions on the effects, the data were analyzed additionally with respect to these factors.

#### Age

**Figure 4:**
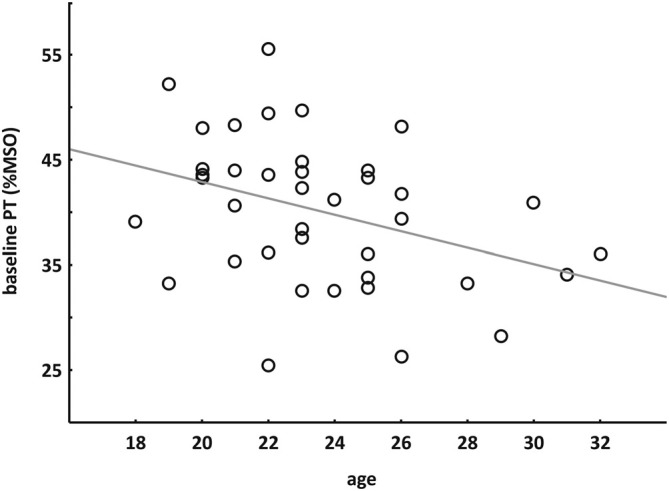
**Correlation between age and baseline PT.** Spearman’s rank correlation indicated that younger subjects had higher PTs.

Spearman’s rank correlation showed a negative correlation between baseline PT (mean session 1 and 2) and age of the subjects (r_s_ = −0.387, *p* = 0.014) indicating that younger subjects had higher PTs (see Figure 4 ).

#### Gender

Pre- and post-TBS PTs of the cTBS group were subjected to an rmANOVA with the between-factor GENDER (m/f) and the within factors SESSION (high/low visual demand) and TIME (pre, post1, post2). There was a main effect of GENDER (*F*_(1, 18)_ = 6.330, *p* = 0.022) indicating that males had significant higher PTs than females. No main effects were found for SESSION (*F*_(1, 18)_ = 0.98, *p* = 0.34) as well as for TIME (*F*_(2, 36)_ = 2.29, *p* = 0.12). The SESSION × TIME interaction was significant (*F*_(2, 36)_ = 4.747, *p* = 0.015), similar to the main analysis reported above without the factor GENDER, but there was no effect in the omnibus interaction. A comparable analysis of the iTBS group data revealed no main effects and no interactions.

Due to the main effect of GENDER in the cTBS group, pre PT values of all subjects (mean baseline values of both sessions) were analyzed with respect to gender differences. Although not statistically significant (*t* = 1.735, *p* = 0.091), there was a trend towards higher PTs in males compared to females. The gender difference is likely to be an artifact of the age differences observed since an analysis of covariance revealed that the trend disappeared (*F*_(1.37)_ = 1.24, *p* = 0.27). Mean age of the male subjects (22.6 ± 3.3 years) was lower than that of the female subjects (24.5 ± 3.0 years) as revealed by the analysis (*F*_(1, 37)_ = 4.01, *p* = 0.053).

#### Order

In the main analysis, we showed that there was no difference in baseline PTs according to the sessions with high and low visual demand. Additionally, we analyzed whether there is an order effect from day 1 to day 2, independent of session type. A paired *t*-test comparing the pre-TBS PT values of the subject’s first session with those of the second session again revealed stability in PT (*t*_(39)_ = 1.484, *p* = 0.146).

## Discussion

In this study, we examined the effects of TBS on visual cortex excitability and investigated if enhanced visual demand following TBS might modulate the effects. Our hypotheses were: (a) TBS will modify PT; (b) the modulation of PT will last for some time if subjects have low visual demand following TBS; and (c) the modulation will be affected if subjects have high visual demand following TBS. The first two hypotheses have to be rejected, because there was no modulation of PT directly after TBS. For cTBS, we found significantly increased PTs following high visual demand after stimulation. Thus, our third hypothesis can be partially accepted. Additionally, there was a negative correlation between baseline PT and age of the subjects.

Several studies showed modulated visual cortex excitability following a variety of rTMS protocols. Conventional 1 Hz rTMS increased PT (Boroojerdi et al., 2000b; Brighina et al., 2002; Fierro et al., 2005), whereas decreased PTs were found after high-frequency rTMS protocols (Fierro et al., 2005; Lang et al., 2007). The direction of the observed effects was similar to the effects in the motor system: whereas low-frequency rTMS usually resulted in decreased MEP amplitudes, higher frequencies were often shown to enhance cortical activity (Fitzgerald et al., 2006). With the introduction of the TBS pattern (Huang et al., 2005), the idea came up of simply replacing the conventional rTMS protocols by TBS. Since cTBS requires shorter application times and weaker stimulation intensities, showing more robust effects on motor cortex excitability compared to 1 Hz rTMS, TBS seems to be the better choice both in terms of efficiency and side effects. The initial inhibitory effect of cTBS as well as the facilitatory effect of iTBS in the motor system have been replicated successfully in quite a few studies (Agostino et al., 2008; Di Lazzaro et al., 2008; Gentner et al., 2008; Suppa et al., 2008; Zafar et al., 2008; Gamboa et al., 2010; Huang et al., 2010). Furthermore, TBS effects were transferred to other cortical regions. For instance, cTBS as well as 1 Hz rTMS increased reaction times in a lexical decision task when applied to the left superior temporal cortex (Brückner et al., 2013). Applied to the frontal eye field, both cTBS and 1 Hz rTMS had inhibitory effects on saccade triggering (Nyffeler et al., 2006). In the visual system, PTs increased following cTBS (Franca et al., 2006) comparable to the observation after 1 Hz rTMS (e.g., Fierro et al., 2005).

However, the initial effects of TBS in the motor system (cTBS inhibitory, iTBS facilitatory) could not always be replicated. For instance, cTBS was shown to not reliably depress all regions of the motor cortex (Martin et al., 2006). In the visual system, no effect of iTBS on PTs was found (Franca et al., 2006). Neither cTBS nor iTBS applied with various stimulation intensities modulated peripheral visual acuity (Brückner and Kammer, 2014). In the present study, neither cTBS nor iTBS had direct effects on PTs. As we observed a dose-dependent effect with cTBS in the language domain (Brückner et al., 2013), TBS applied with 100% of individual PT was supposed to have a stronger modulatory effect than applied with 80% PT intensity (Franca et al., 2006). Surprisingly, we observed no modulation of PTs. Therefore, it is conceivable that cTBS directly modulates PT only at subthreshold intensity levels, whereas iTBS seems not to be able to modulate visual cortex excitability at all.

In the last years, the initial cortical activation state of the neurons and the ongoing brain activity during brain stimulation has received more and more attention. State dependency of TMS effects were described in many cortical areas. In the motor system, contraction of the target muscle immediately after TBS led to facilitation of cortical activity, independent of whether cTBS or iTBS was applied (Huang et al., 2008). Preconditioning motor cortex excitability by transcranial direct current stimulation reversed the conditioning effects of 1 Hz rTMS (Siebner et al., 2004). In the visual cortex, manipulation of the initial state of the neurons by selective adaptation to colored stimuli showed that TMS facilitated the less active neuron population (Silvanto et al., 2007). Several other studies showed evidence for homeostatic metaplasticity in the human cortex (Müller-Dahlhaus and Ziemann, 2015). It was shown that visual demand influences cortical excitability *per se*: Rauschecker et al. (2004) demonstrated that PTs increased with higher luminance contrast levels. They concluded that an increase in contrast causes higher overall activation of the neurons and may mask the activation produced by TMS pulses. However, they measured PT during visual demand. The visual task in our study was applied between two PT measurements. One could speculate that an increase of neuronal activity during the Landolt C optotype task persists during the subsequent PT measurement, resulting in higher PTs. If this would be the case, we would expect higher PTs following visual demand in the iTBS group, too. But PTs were unchanged in both sessions following iTBS—with or without high visual demand. PTs increased only after high visual demand preceded by cTBS, indicating that the combination of this TBS pattern with the following visual task modulates cortical excitability. According to the stochastic resonance phenomenon, in a nonlinear system the injection of low-level noise can lower the threshold of the system (Schwarzkopf et al., 2011), whereas at higher intensities TMS should disrupt behavioral performance. Assuming that application of TBS with 100% of individual PT intensity would add low levels of noise to the visual system, this would decrease PTs. Although not statistically significant, we observed numerically lower thresholds following cTBS. It is conceivable that only the combination of cTBS and high visual demand caused by the following visual acuity task generates noise levels high enough to impair phosphene detection. Thus, stochastic resonance could explain our TBS effects in some respects. However, this would not explain the increased PTs following subthreshold cTBS observed by Franca et al. (2006) and Allen et al. (2014), as well as the lack of effects applying iTBS. As, to our knowledge, the previous studies estimating the stochastic resonance phenomenon focused on online TMS procedures (Miniussi et al., 2013), it is conceivable that different mechanisms may underlie the effects of offline TMS protocols.

Recently, it was shown that cTBS applied to the visual cortex enhanced conscious vision despite of decreased cortical excitability (Allen et al., 2014). This counterintuitive finding suggested that cTBS might increase cortical inhibition, leading to a higher signal-to-noise ratio and thus better performance in a conscious perception task. In contrast, cTBS decreased accuracy and confidence in a perceptual discrimination task (Rahnev et al., 2013), probably by reducing resting state connectivity between visual areas. Allen et al. (2014) argued that the discrepancy between their results and those of Rahnev et al. (2013) is explained by stimulation intensity. They stated that slight increases of inhibition caused by cTBS can be conducive to detection tasks, but if inhibition is increased to a greater extend due to higher cTBS intensity, this may led to reduced detection capacity (Allen et al., 2014). As our stimulation intensity was 100% of individual PT and thus still higher than the 80% PT intensity applied by Rahnev et al. (2013), following the argument of Allen et al. (2014), in our study detection capacity should be strongly impaired. However, our visual task was not designed to analyze the change of visual acuity, since we found no changes of visual acuity following TBS applied with various intensities (Brückner and Kammer, 2014).

A possible explanation of our data is that the effect of cTBS applied with 100% of individual PT intensity only emerges if enhanced activity of the neurons is requested following stimulation. This could explain why no direct effect of stimulation on PT was observed, but an effect manifested following high visual demand. As a second hypothesis following Rahnev et al. (2013), cTBS reduces resting state connectivity between visual areas. A subsequent visual task thus requires neural activity to be increased compared to the normal state, in order to compensate for the stimulation effect. This higher activity might then mask the neuronal activation through TMS pulses (Rauschecker et al., 2004) and thus increases PTs not only during but also following visual demand. In this case, the observed absence of any iTBS effect has to be interpreted in an alternative way. It is conceivable that iTBS might increase resting state connectivity, making it easier to perform the visual acuity task without affecting the neuronal activation level and without modulating PTs. A third possible explanation is that the visual task itself modulates cortical excitability as demonstrated by modified PTs (Rauschecker et al., 2004; Antal et al., 2014). Following this view, our data would indicate that iTBS protects against the inhibitory effect of the task and that cTBS has no modulatory effect. Of course, all these interpretations are highly speculative, and due to methodological differences between the studies a direct comparison is impossible. For instance, Rahnev et al. (2013) applied cTBS to the occipital cortex with the subject’s eyes closed. Since activation during stimulation is an important factor for rTMS effects (Silvanto et al., 2007), the initial state of the neurons in our study might be different from that in their study. In comparison with both studies (Rahnev et al., 2013; Allen et al., 2014), our stimulation intensity was higher and our experimental setup was different, including stimulator, coil, and PT determination procedure, making it difficult to compare the results of the studies.

Beside the effects of TBS and visual demand on visual cortex excitability, our study revealed some additional results. We observed a high test-retest-reliability of baseline PT values. Due to the between-subject design, mean PT values differed between the two groups investigated. Comparing the baseline PTs of the subjects across two sessions and two measurements per session, there was no difference on any condition. This stability is in line with the results from previous studies (Stewart et al., 2001; Boroojerdi et al., 2002; Antal et al., 2003, 2004; Siniatchkin et al., 2011), confirming PT as a reliable marker of visual cortex excitability.

In our additional analyses, we observed a negative correlation between baseline PT and age, indicating that younger subjects had higher PTs. In the motor cortex, some studies observed no correlation between age of the subjects and motor threshold (Rossi et al., 2004; Oliviero et al., 2006), whereas others showed that motor threshold might increase with age (Rossini et al., 1992; Peinemann et al., 2001). However, in these studies age range of the subjects was much higher (e.g., 20–38 years in the young group and 48–71 years in the old group in Peinemann et al., 2001). As age range was 18–32 years in the present study, all subjects would belong to the young group of the studies mentioned above. In a TMS study on children, significantly higher resting motor thresholds were found compared to adult controls (Bender et al., 2005). Motor thresholds in children decreased with age until reaching an adult level at age 13 (Nezu et al., 1997), indicating a maturation process. From that we could speculate, that the observed decrease of PT with age might indicate a prolonged maturation process of the visual system. Finally, we observed a trend to higher PTs in males compared to females, whereas most studies showed no gender differences in baseline cortex excitability (Livingston et al., 2010; Perciavalle et al., 2010; Cuypers et al., 2014). In line with our observation that younger subjects had higher PTs, this difference is most probably due to the fact that mean age of males was lower than that of females, as revealed by the analysis of covariance.

In summary, two main findings can be derived from our results:

(a) The role of stimulation intensity applying TBS.

We observed no direct effect on visual cortex excitability using 100% of individual PT. Since two independent studies in Franca et al. (2006) and Allen et al. (2014) showed increased PTs following cTBS applied with 80% PT intensity, the lack of modulation is likely to arise from stimulation intensity. Compared to cTBS effects on posterior temporal cortex (Brückner et al., 2013), no dose-dependency in visual cortex excitability was observed.

(b) The role of controled neuronal activity not only before or during but also following TBS.

It is well-known that the initial state of the neurons plays an important role in modulating brain functions using TMS (Silvanto and Pascual-Leone, 2008). Several studies showed evidence for homeostatic metaplasticity in the human cortex (Müller-Dahlhaus and Ziemann, 2015). Since controled activity in the human motor cortex influenced the effects of prior TBS application (Huang et al., 2008), our study shows similar results with regard to the human visual cortex, confirming the importance of scrupulously controled experimental conditions.

## Conflict of Interest Statement

The authors declare that the research was conducted in the absence of any commercial or financial relationships that could be construed as a potential conflict of interest.

## References

[B1] Agostino R. Lezzi E. Dinapoli L. Suppa A. Conte A. Berardelli A. (2008). Effects of intermittent theta-burst stimulation on practice-related changes in fast finger movements in healthy subjects. Eur. J. Neurosci. 28, 822–828. 10.1111/j.1460-9568.2008.06373.x 18702693

[B2] Allen C. P. G. Dunkley B. T. Muthukumaraswamy S. D. Edden R. Evans C. J. Sumner P.. (2014). Enhanced awareness followed reversible inhibition of human visual cortex: a combined TMS, MRS and MEG study. PLos One 9:e100350. 10.1371/journal.pone.0100350 24956195 PMC4067303

[B3] Antal A. Ambrus G. G. Chaieb L. (2014). Toward unraveling reading-related modulations of tDCS-induced neuroplasticity in the human visual cortex. Front. Psychol. 5:642. 10.3389/fpsyg.2014.00642 24999339 PMC4064701

[B4] Antal A. Kincses T. Z. Nitsche M. A. Paulus W. (2003). Manipulation of phosphene thresholds by transcranial direct current stimulation in man. Exp. Brain Res. 150, 375–378. 10.3410/f.1013621.191430 12698316

[B5] Antal A. Kincses T. Z. Nitsche M. A. Bartfai O. Paulus W. (2004). Excitability changes induced in the human primary visual cortex by transcranial direct current stimulation: direct electrophysiological evidence. Invest. phthalmol. Vis. Sci. 45, 702–707. 10.1167/iovs.03-0688 14744917

[B6] Aurora S. K. Welch K. M. A. Al-Sayed F. (2003). The threshold for phosphenes is lower in migraine. Cephalalgia 23, 258–263. 10.1046/j.1468-2982.2003.00471.x 12716342

[B7] Bach M. (1996). The Freiburg Visual Acuity test—automatic measurement of visual acuity. Optom. Vis. Sci. 73, 49–53. 10.1097/00006324-199601000-00008 8867682

[B8] Barker A. T. Freeston I. L. Jalinous R. Merton P. A. Morton H. B. (1985). Magnetic stimulation of the human brain. J. Physiol-London 369, P3–P3.

[B9] Bender S. Basseler K. Sebastian I. Resch F. Kammer T. Oelkers-Ax R. (2005). Transcranial magnetic stimulation evokes giant inhibitory potentials in children. Ann. Neurol. 58, 58–67. 10.1002/ana.20521 15984026

[B10] Bocci T. Caleo M. Tognazzi S. Francini N. Briscese L. Maffei L.. (2014). Evidence for metaplasticity in the human visual cortex. J. Neural Transm. 121, 221–231. 10.1007/s00702-013-1104-z 24162796

[B11] Boroojerdi B. Bushara K. O. Corwell B. Immisch I. Battaglia F. Muellbacher W.. (2000a). Enhanced excitability of the human visual cortex induced by short-term light deprivation. Cereb. Cortex 10, 529–534. 10.1093/cercor/10.5.529 10847602

[B13] Boroojerdi B. Prager A. Muellbacher W. Cohen L. G. (2000b). Reduction of human visual cortex excitability using 1-Hz transcranial magnetic stimulation. Neurology 54, 1529–1531. 10.1212/wnl.54.7.1529 10751273

[B12] Boroojerdi B. Meister I. G. Foltys H. Sparing R. Cohen L. G. Topper R. (2002). Visual and motor cortex excitability: a transcranial magnetic stimulation study. Clin. Neurophysiol. 113, 1501–1504. 10.1016/s1388-2457(02)00198-0 12169333

[B14] Brighina F. Piazza A. Daniele O. Fierro B. (2002). Modulation of visual cortical excitability in migraine with aura: effects of 1 Hz repetitive transcranial magnetic stimulation. Exp. Brain Res. 145, 177–181. 10.1007/s00221-002-1096-7 12110957

[B15] Brückner S. Kammer T. (2014). Is theta burst stimulation applied to visual cortex able to modulate peripheral visual acuity? PLoS One 9:e99429. 10.1371/journal.pone.0099429 24914682 PMC4051767

[B16] Brückner S. Kiefer M. Kammer T. (2013). Comparing the after-effects of continuous theta burst stimulation and conventional 1Hz rTMS on semantic processing. Neuroscience 233, 64–71. 10.1016/j.neuroscience.2012.12.033 23276670

[B17] Cuypers K. Thijs H. Meesen R. L. J. (2014). Optimization of the transcranial magnetic stimulation protocol by defining a reliable estimate for corticospinal excitability. PLoS One. 9:e86380. 10.1371/journal.pone.0086380 24475111 PMC3901672

[B18] Di Lazzaro V. Pilato F. Dileone M. Profice P. Oliviero A. Mazzone P.. (2008). The physiological basis of the effects of intermittent theta burst stimulation of the human motor cortex. J. Physiol. 586, 3871–3879. 10.1113/jphysiol.2008.152736 18566003 PMC2538925

[B19] Fierro B. Brighina F. Vitello G. Piazza A. Scalia S. Giglia G.. (2005). Modulatory effects of low- and high-frequency repetitive transcranial magnetic stimulation on visual cortex of healthy subjects undergoing light deprivation. J. Physiol. 565, 659–665. 10.1113/jphysiol.2004.080184 15760946 PMC1464536

[B20] Fitzgerald P. B. Fountain S. Daskalakis Z. J. (2006). A comprehensive review of the effects of rTMS on motor cortical excitability and inhibition. Clin. Neurophysiol. 117, 2584–2596. 10.1016/j.clinph.2006.06.712 16890483

[B21] Fleming M. K. Sorinola I. O. Newham D. J. Roberts-Lewis S. F. Bergmann J. H. M. (2012). The effect of coil type and navigation on the reliability of transcranial magnetic stimulation. IEEE Trans. Neural Syst. Rehabil. Eng. 20, 617–625. 10.1109/tnsre.2012.2202692 22695363

[B22] Franca M. Koch G. Mochizuki H. Huang Y. Z. Rothwell J. C. (2006). Effects of theta burst stimulation protocols on phosphene threshold. Clin. Neurophysiol. 117, 1808–1813. 10.1016/j.clinph.2006.03.019 16797230

[B23] Gamboa O. L. Antal A. Moliadze V. Paulus W. (2010). Simply longer is not better: reversal of theta burst after-effect with prolonged stimulation. Exp. Brain Res. 204, 181–187. 10.1007/s00221-010-2293-4 20567808 PMC2892066

[B24] Gentner R. Wankerl K. Reinsberger C. Zeller D. Classen J. (2008). Depression of human corticospinal excitability induced by magnetic theta-burst stimulation: evidence of rapid polarity-reversing metaplasticity. Cereb. Cortex 18, 2046–2053. 10.1093/cercor/bhm239 18165282

[B25] Huang Y. Z. Edwards M. J. Rounis E. Bhatia K. P. Rothwell J. C. (2005). Theta burst stimulation of the human motor cortex. Neuron 45, 201–206. 10.1016/j.neuron.2004.12.033 15664172

[B26] Huang Y. Z. Rothwell J. C. Edwards M. J. Chen R. S. (2008). Effect of physiological activity on an NMDA-dependent form of cortical plasticity in human. Cereb. Cortex 18, 563–570. 10.1093/cercor/bhm087 17573373

[B27] Huang Y. Z. Rothwell J. C. Lu C. S. Chuang W. L. Lin W. Y. Chen R. S. (2010). Reversal of plasticity-like effects in the human motor cortex. J. Physiol. 588, 3683–3693. 10.1113/jphysiol.2010.191361 20660564 PMC2997480

[B28] Kammer T. Baumann L. W. (2010). Phosphene thresholds evoked with single and double TMS pulses. Clin. Neurophysiol. 121, 376–379. 10.1016/j.clinph.2009.12.002 20079689

[B29] Kammer T. Beck S. (2002). Phosphene thresholds evoked by transcranial magnetic stimulation are insensitive to short-lasting variations in ambient light. Exp. Brain Res. 145, 407–410. 12136391 10.1007/s00221-002-1160-3

[B30] Kammer T. Beck S. Erb M. Grodd W. (2001a). The influence of current direction on phosphene thresholds evoked by transcranial magnetic stimulation. Clin. Neurophysiol. 112, 2015–2021. 10.1016/s1388-2457(01)00673-3 11682339

[B31] Kammer T. Beck S. Thielscher A. Laubis-Herrmann U. Topka H. (2001b). Motor thresholds in humans: a transcranial magnetic stimulation study comparing different pulse waveforms, current directions and stimulator types. Clin. Neurophysiol. 112, 250–258. 10.1016/s1388-2457(00)00513-7 11165526

[B32] Kammer T. Puls K. Erb M. Grodd W. (2005). Transcranial magnetic stimulation in the visual system. II. Characterization of induced phosphenes and scotomas. Exp. Brain Res. 160, 129–140. 10.1007/s00221-004-1992-0 15368087

[B33] Kammer T. Vorwerg M. Herrnberger B. (2007). Anisotropy in the visual cortex investigated by neuronavigated transcranial magnetic stimulation. Neuroimage 36, 313–321. 10.1016/j.neuroimage.2007.03.001 17442592

[B34] Lang N. Siebner H. R. Chadaide Z. Boros K. Nitsche M. A. Rothwell J. C.. (2007). Bidirectional modulation of primary visual cortex excitability: a combined tDCS and rTMS study. Invest. Ophthalmol. Vis. Sci. 48, 5782–5787. 10.1167/iovs.07-0706 18055832

[B35] Livingston S. C. Goodkin H. P. Ingersoll C. D. (2010). The influence of gender, hand dominance and upper extremity length on motor evoked potentials. J. Clin. Monit. Comput. 24, 427–436. 10.1007/s10877-010-9267-8 21110222

[B36] Lou A. R. Madsen K. H. Paulson O. B. Julian H. O. Prause J. U. Siebner H. R.. (2011). Monocular visual deprivation suppresses excitability in adult human visual cortex. Cereb. Cortex 21, 2876–2882. 10.1093/cercor/bhr082 21531780

[B37] Marg E. Rudiak D. (1994). Phosphenes induced by magnetic stimulation over the occipital brain—description and probable site of stimulation. Optom. Vis. Sci. 71, 301–311. 10.1097/00006324-199405000-00001 8065706

[B38] Martin P. G. Gandevia S. C. Taylor J. L. (2006). Theta burst stimulation does not reliably depress all regions of the human motor cortex. Clin. Neurophysiol. 117, 2684–2690. 10.1016/j.clinph.2006.08.008 17029949

[B39] Meyer B. U. Diehl R. Steinmetz H. Britton T. C. Benecke R. (1991). Magnetic stimuli applied over motor and visual cortex: influence of coil position and field polarity on motor responses, phosphenes, and eye movements. Electroencephalogr. Clin. Neurophysiol. Suppl. 43, 121–134. 1773752

[B40] Miniussi C. Harris J. A. Ruzzoli M. (2013). Modelling non-invasive brain stimulation in cognitive neuroscience. Neurosci. Biobehav. R. 37, 1702–1712. 10.1016/j.neubiorev.2013.06.014 23827785

[B41] Müller-Dahlhaus F. Ziemann U. (2015). Metaplasticity in human cortex. Neuroscientist 21, 185–202. 10.1177/1073858414526645 24620008

[B42] Nezu A. Kimura S. Uehara S. Kobayashi T. Tanaka M. Saito K. (1997). Magnetic stimulation of motor cortex in children: maturity of corticospinal pathway and problem of clinical application. Brain Dev. 19, 176–180. 10.1016/s0387-7604(96)00552-9 9134188

[B43] Nyffeler T. Wurtz P. Luscher H. R. Hess C. W. Senn W. Pflugshaupt T.. (2006). Repetitive TMS over the human oculomotor cortex: comparison of 1-Hz and theta burst stimulation. Neurosci. Lett. 409, 57–60. 10.1016/j.neulet.2006.09.011 17049743

[B44] Oliviero A. Profice P. Tonali P. A. Pilato F. Saturno E. Dileone M.. (2006). Effects of aging on motor cortex excitability. Neurosci. Res. 55, 74–77. 10.1016/j.neures.2006.02.002 16584795

[B45] Peinemann A. Lehner C. Conrad B. Siebner H. R. (2001). Age-related decrease in paired-pulse intracortical inhibition in the human primary motor cortex. Neurosci. Lett. 313, 33–36. 10.1016/s0304-3940(01)02239-x 11684333

[B46] Peirce J. W. (2007). PsychoPy - Psychophysics software in Python. J. Neurosci. Meth. 162, 8–13. 10.1016/j.jneumeth.2006.11.017 17254636 PMC2018741

[B47] Perciavalle V. Coco M. Alagona G. Maci T. Perciavalle V. (2010). Gender differences in changes of motor cortex excitability during elevated blood lactate levels. Somatosens. Mot. Res. 27, 106–110. 10.3109/08990220.2010.507102 20704473

[B48] Rahnev D. Kok P. Munneke M. Bahdo L. De Lange F. P. Lau H. (2013). Continuous theta burst transcranial magnetic stimulation reduces resting state connectivity between visual areas. J. Neurophysiol. 110, 1811–1821. 10.1152/jn.00209.2013 23883858

[B49] Rauschecker A. M. Bestmann S. Walsh V. Thilo K. V. (2004). Phosphene threshold as a function of contrast of external visual stimuli. Exp. Brain Res. 157, 124–127. 10.1007/s00221-004-1910-5 15164153

[B50] Rossi S. Miniussi C. Pasqualetti P. Babiloni C. Rossini P. M. Cappa S. F. (2004). Age-related functional changes of prefrontal cortex in long-term memory: A repetitive transcranial magnetic stimulation study. J. Neurosci. 24, 7939–7944. 10.3410/f.1021134.241471 15356207 PMC6729939

[B51] Rossini P. M. Desiato M. T. Caramia M. D. (1992). Age-related-changes of motor evoked-potentials in healthy humans - noninvasive evaluation of central and peripheral motor tracts excitability and conductivity. Brain Res. 593, 14–19. 10.1016/0006-8993(92)91256-e 1458317

[B52] Sandrini M. Umiltà C. Rusconi E. (2011). The use of transcranial magnetic stimulation in cognitive neuroscience: A new synthesis of methodological issues. Neurosci. Biobehav. Rev. 35, 516–536. 10.1016/j.neubiorev.2010.06.005 20599555

[B53] Schwarzkopf D. S. Silvanto J. Rees G. (2011). Stochastic resonance effects reveal the neural mechanisms of transcranial magnetic stimulation. J. Neurosci. 31, 3143–3147. 10.1523/jneurosci.4863-10.2011 21368025 PMC3059801

[B54] Siebner H. R. Lang N. Rizzo V. Nitsche M. A. Paulus W. Lemon R. N.. (2004). Preconditioning of low-frequency repetitive transcranial magnetic stimulation with transcranial direct current stimulation: evidence for homeostatic plasticity in the human motor cortex. J. Neurosci. 24, 3379–3385. 10.1523/jneurosci.5316-03.2004 15056717 PMC6730024

[B55] Silvanto J. Pascual-Leone A. (2008). State-dependency of transcranial magnetic stimulation. Brain Topogr. 21, 1–10. 10.1007/s10548-008-0067-0 18791818 PMC3049188

[B56] Silvanto J. Muggleton N. G. Cowey A. Walsh V. (2007). Neural adaptation reveals state-dependent effects of transcranial magnetic stimulation. Eur. J. Neurosci. 25, 1874–1881. 10.1111/j.1460-9568.2007.05440.x 17408427

[B57] Siniatchkin M. Schlicke C. Stephani U. (2011). Transcranial magnetic stimulation reveals high test-retest reliability for phosphenes but not for suppression of visual perception. Clin. Neurophysiol. 122, 2475–2481. 10.1016/j.clinph.2011.05.003 21641863

[B58] Stewart L. M. Walsh V. Rothwell J. C. (2001). Motor and phosphene thresholds: a transcranial magnetic stimulation correlation study. Neuropsychologia 39, 415–419. 10.1016/s0028-3932(00)00130-5 11164880

[B59] Suppa A. Ortu E. Zafar N. Deriu F. Paulus W. Berardelli A.. (2008). Theta burst stimulation induces after-effects on contralateral primary motor cortex excitability in humans. J. Physiol. 586, 4489–4500. 10.1113/jphysiol.2008.156596 18669534 PMC2614023

[B60] Wichmann F. A. Hill N. J. (2001). The psychometric function: I. Fitting, sampling and goodness of fit. Percept. Psychophys. 63, 1293–1313. 10.3758/bf03194544 11800458

[B61] Zafar N. Paulus W. Sommer M. (2008). Comparative assessment of best conventional with best theta burst repetitive transcranial magnetic stimulation protocols on human motor cortex excitability. Clin. Neurophysiol. 119, 1393–1399. 10.1016/j.clinph.2008.02.006 18400556

